# A Case Series Analysis of Major Vascular Revascularization

**DOI:** 10.7759/cureus.27595

**Published:** 2022-08-02

**Authors:** Rajeshwar Yadav, Swati Pathak, Satisha Hegde

**Affiliations:** 1 Department of Cardiovascular and Thoracic Surgery, Institute of Medical Sciences, Banaras Hindu University, Varanasi, IND; 2 Department of Pathology, Jawaharlal Institute of Postgraduate Medical Education and Research (JIPMER), Puducherry, IND

**Keywords:** peripheral arterial disease (pad), cavo-atrial shunt, pta, carotid endarterectomy, aortofemoral bypass, revascularization, aortoiliac, aorta

## Abstract

Purpose

Atherosclerosis is a generalized disorder and can begin to develop in the abdominal aorta by the second decade of life. The nature of these lesions in coronaries and aorta is atheromatous and less sclerotic when compared to peripheral arteries. A broad spectrum of presentations and different types of lesions demand a personalized approach for the best outcome. This study is a case series analysis of major vascular revascularization. We aim to study various revascularization surgeries and underline the wide range of vascular lesions to which it is applied.

Methods

This is a study based on accrual patient records of all major vascular revascularization surgical/interventional procedures conducted at a tertiary care center for one year.

Results

A total of 110 patients were operated on for vascular diseases. Among these, 86 (78.81%) were men, and 24 (21.81%) were women. The femoropopliteal segment (n=47) was most commonly involved, followed by the common carotid artery (n=20). Atherosclerosis was the main cause of vascular occlusion (81.8%), followed by aneurysm of the aorta (14.5%) and coarctation of the aorta (2.7%). Smoking (62.2%) accounted to be the leading risk factor, followed by hypertension, diabetes, and hyperlipidemia. The majority of patients had a good outcome (92.7%). Minor complications (7.3%) include seroma formation and wound infection, which were managed conservatively. The repair was performed by autologous vein graft in 30% of patients and by synthetic polytetrafluoroethylene (PTFE) graft in 70% of patients. Carotid artery stenting was the most common endovascular procedure performed (n=5). Femoropopliteal bypass grafting was the most common procedure, followed by carotid endarterectomy (n=20) and aortofemoral bypass (n=14).

Conclusion

The application of novel techniques such as cavo-atrial shunt in Budd-Chiari syndrome calls attention to the broadened scope of vascular surgery, and the modification of the conventional method of the carotid endarterectomy underscores the evolution of vascular revascularization. Our study thus highlighted that a wide spectrum of vascular lesions ranging from carotid artery stenosis to extensive below-knee disease, either atherosclerotic or aneurysmal, can be successfully treated with surgical revascularization techniques.

## Introduction

In 1814, Robert Graham (physician) first described aortoiliac disease. It was a century later in 1923 when French surgeon René Leriche explained the condition as a thrombotic lesion of the distal part of the aorta associated with a triad of claudication, impotence, and decreased peripheral pulses. This brought into the light a broad spectrum of atherosclerotic cardiovascular diseases that affect coronary, visceral, aortoiliac, and infrainguinal vascular beds. Patients with coronary artery disease may have associated peripheral vascular disease, carotid artery stenosis, and renal artery stenosis. The aging population, increase in smoking, diabetes mellitus, hypertension, and obesity are accountable for the worldwide rise in its prevalence [1-3]. The measurement of incidence and prevalence of this condition is not easy to estimate as many patients are asymptomatic due to the formation of a collateral system. In India, atherosclerosis and Takayasu arteritis is the etiology in 96% of aortoiliac diseases [4]. In population- and epidemiology-based studies, it is estimated that 20%-30% of diabetic patients over 65 years of age have peripheral arterial disease (PAD) [5]. Certain studies reveal that the ratio of symptomatic to asymptomatic peripheral arterial disease (PAD) is around 1:3 [6].

## Materials and methods

This study is a retrospective study based on accrual records of patients who had undergone major vascular surgical/interventional procedures at a tertiary care center in India for one year. Ethical approval was taken from the board of the institute. The diagnosis was made by full physical examination and clinical assessment (ankle-brachial index (ABI) measured), Doppler study, and cardiac computed tomography (CT) scan/CT angiography/arterial angiography.

Inclusion criteria

The study was conducted for a duration of one year. The study includes all patients who were admitted for aortic/arterial aneurysmal disease. All patients who had an aortic or aortoiliac occlusive disease (AIOD) and peripheral arterial disease not relieved by medical conservative management with rest pain and/or chronic limb-threatening ischemia and undergoing graft bypass surgery for treatment/palliation (Budd-Chiari syndrome) were included.

Exclusion criteria

We have not included minor vascular procedures in this study, which did not require the application of graft. All peripheral arterial disease patients relieved by medical management were also not included.

Patients were operated on under general anesthesia with/without cardiopulmonary bypass (CPB), regional anesthesia, or nerve block. Routine use of intravenous unfractionated heparin was done just before clamping the vessels, followed by intravenous sodium bicarbonate just before releasing the clamps after anastomosis. The successful repair was assessed by the return of distal pulsation at the end of the operation. All patients received intravenous preoperative, prophylactic antibiotics, which were continued postoperatively for 5-7 days according to need. All patients received intravenous heparin initial dose of 5,000 units and then 25,000 units with dextran (low molecular weight: 40,000) continuous infusion drip every 24 hours for three days postoperatively along with oral aspirin (375 mg) and warfarin (2 mg). Patients were routinely followed at one, three, and six months after discharge in the outpatient department, and status was assessed by taking history, doing a physical examination, measuring ankle-brachial index (ABI), INR monitoring, and performing imaging studies.

The types of surgery/endovascular procedures performed are listed in Table 1 and Table 2.

**Table 1:** Surgical revascularization procedures

Surgical procedures/disease	Number of patients	Outcome
Ascending to descending aorta bypass graft under CPB in complete interruption of arch distal to the left subclavian artery	3	Excellent
Inter-positional synthetic graft in infrarenal aortic aneurysm not involving common iliac vessels	6	Excellent
Aortobifemoral grafting in infrarenal aortic aneurysm extending up to bilateral common iliac vessels	8	Excellent
Extraperitoneal thoracobifemoral bypass grafting	5	Good outcome (n=4), seroma formation (n=1 ) managed conservatively
Carotid endarterectomy	20	Excellent
Cavo-atrial shunt for Budd-Chiari syndrome	1	Excellent
Right subclavian to brachial bypass in huge subclavian artery aneurysm	1	Wound infection managed conservatively
Left subclavian to right axillary bypass	3	Excellent
Axillo-radial bypass	3	Good outcome (n=2), wound infection (n=1) managed conservatively
Femoro-femoral crossover bypass	10	Excellent (n=8), seroma formation (n=2) managed conservatively
Common femoral to distal femoral bypass	12	Excellent (n=11), seroma formation (n=1) managed conservatively
Femoropopliteal bypass	15	Excellent
Femorotibial bypass	10	Excellent (n=9), seroma formation (n=1) managed conservatively
Axillo-bifemoral (extra-anatomical ) bypass	2	Excellent

**Table 2:** Percutaneous endovascular procedures

Percutaneous interventions/disease	Number of patients	Outcome
Stenting for occlusion of the suprarenal aorta	2	Excellent
Stenting for abdominal infrarenal aortic aneurysm	1	Conversion to surgical bypass grafting
Unilateral renal artery stenting	1	Excellent
Subclavian artery stenting	2	Excellent
Carotid artery stenting	5	Excellent

Various vascular lesions observed and interventions applied are described in Table 3 with a brief description of the procedures.

**Table 3:** Lesions and procedures performed

Disease	Total patients	Surgical revascularization	Percutaneous interventions
Carotid artery stenotic/near-total occlusive lesions	25	20	5
Coarctation of the aorta	3	3	0
Aortic aneurysm/atheroslerotic lesion not involving common iliac vessels	7	6	1 (converted to inter-positional bypass graft)
Abdominal aortic aneurysm/atheroslerotic lesion involving common iliac vessels	7	7	0
Subclavian artery aneurysm/atheroslerotic lesion	6	4	2
Axillary artery stenotic/occlusive lesion	1	1	0
Brachial artery aneurysm atheroslerotic lesion	2	2	0
Suprarenal aorta block	3	1	2
Unilateral renal artery stenosis	1	0	1
Femoral artery stenosis/occlusive/aneurysm	22	22	0
Below-knee bypass	25	25	0
Extra-anatomical bypass for thoracic aorta stenotic/occlusive lesions	7	7	0
Cavo-atrial shunt for Budd-Chiari syndrome	1	1	0
Total	110	99	11

The following cases are examples of surgically managed patients in which different surgical repairs and anastomoses were performed.

A 36-year-old male with complete interruption of arch distal to the left subclavian artery underwent surgical correction by ascending to descending aorta bypass with a 20-mm tubular polytetrafluoroethylene (PTFE) graft. Following median sternotomy and cannulation, the descending aorta is accessed by opening the posterior pericardial reflection. The graft was carefully placed anterior to the inferior pulmonary vein from behind the inferior vena cava (IVC), avoiding compression on the right atrium (RA) and ventricle. The anastomosis was performed using 4-0 polypropylene sutures. Inotropic support was required in the immediate postoperative period to counter the sudden reduction in the afterload.

A 65-year-old male was diagnosed with a case of an infrarenal abdominal aortic aneurysm involving common iliac vessels. Aortobifemoral grafting with a Y-shaped tubular PTFE graft measuring 22×11 mm was done along with aneurysm repair. The aortic aneurysm was accessed transperitoneal by laparotomy incision. The bilateral femoral artery was explored via longitudinal groin incisions. The clamp was placed below the renal artery, and proximal anastomosis was done in an end-to-end fashion using a 3-0 polypropylene suture. The Y limbs of the graft were brought down in the groin region by creating a tunnel. The end-to-end distal anastomosis was completed using a 5-0 polypropylene suture. Correct placement of the clamps and avoidance of injury to adjacent structures while forming tunnels prevented hazards postoperatively.

In the case of a 20-year-old female, the long segment of the suprarenal aorta was blocked. Descending aorta to bifemoral artery bypass (extraperitoneal) was done using an 18×9 mm PTFE graft. A Y graft with long limbs was used. Via left thoracotomy incision, end-to-side proximal anastomosis was done, and the limb was tunneled down to the left hypochondriac region where a subcostal incision was performed. From there, the graft was negotiated extraperitoneally to reach the bilateral groin regions to complete the distal anastomosis in an end-to-side fashion.

**Figure 1:**
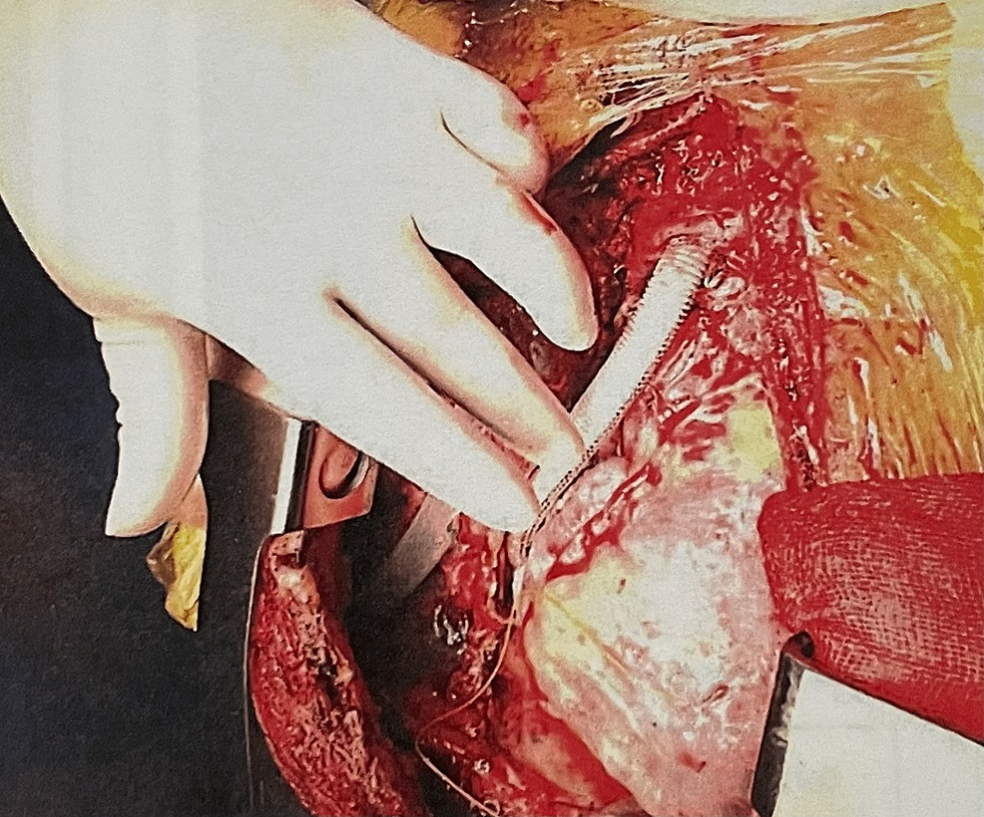
Cavo-atrial shunt for Budd-Chiari syndrome using an 18-mm polytetrafluoroethylene tubular graft

A 50-year-old male with Budd-Chiari syndrome was operated on via a right transdiaphragmatic thoracotomy from the ninth intercostal space (ICS) (Figure 1). He was surgically corrected using an 18-mm tubular PTFE graft from the right atrium to the inferior vena cava (IVC). He underwent a cavo-atrial shunt via extended right anterolateral thoracotomy in the seventh ICS crossing the costal arch. The liver was mobilized carefully all around the diaphragm and retracted anteriorly. With meticulous dissection, the IVC was identified, and a segment of the IVC between the hepatic vein and the right renal vein was isolated. One end of the 18-mm PTFE graft was anastomosed to the IVC using a 6-0 polypropylene suture. Then, the graft was passed into the thorax from a separate hole made in the diaphragm in the paravertebral region. The pericardium was opened, and the graft was anastomosed on the free wall of the RA using a 5-0 polypropylene suture.

**Figure 2:**
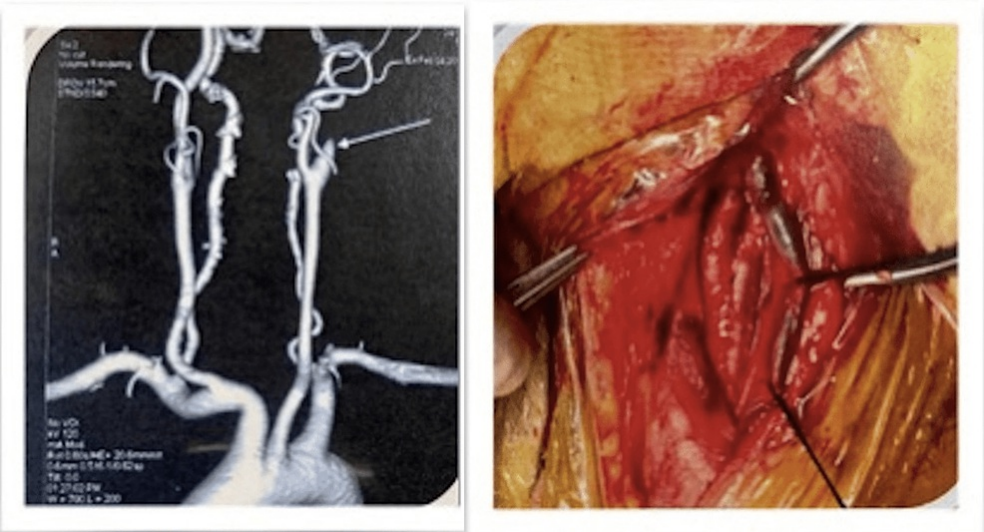
Carotid endarterectomy (preoperative angiography image (left) and intraoperative image (right))

The next case is that of a 48-year-old female with left internal carotid artery occlusion (Figure 2). Carotid endarterectomy with saphenous venous patch repair was done. Conventionally, to maintain cerebral circulation, shunts are used, which also bypasses the blood from the operative site. Nontraumatic vascular clamps were used without shunting, which not only decreased the operative time but also the need for additional suturing and reinforcement over the artery.

In a 45-year-old male with complete right common iliac artery occlusion with good distal collaterals, femoro-femoral crossover bypass grafting with an 8-mm tubular PTFE graft was done. Tunneling was done before heparinization. The graft was positioned in an inverted C shape. An end-to-side anastomosis was performed using a 5-0 polypropylene suture. The graft should be free from tension or kink all along its course to prevent graft thrombosis or bleeding from the anastomosis site.

A 55-year-old male presented with multiple blocks in the femora-popliteal segment. A femoral-posterior tibial artery bypass with reversed saphenous venous graft (RSVG) was done. Two separate incisions were made, exposing the femoral artery at the groin region and another one to access the tibial artery. In below-knee bypass, the lumen of the target artery is small, and there is a need to provide movement at joints. The discrepancy in the lumen of conduit and target remains the essential cause of graft failure. Considering this, a below-knee bypass saphenous venous graft was used.

A 56-year-old female had a huge pseudoaneurysm arising from the distal subclavian artery. The pseudoaneurysm was excised, and proximal subclavian to brachial artery bypass grafting with an 8-mm tubular PTFE graft was done. An oblique subclavicular incision was made to expose the pseudoaneurysm, which was excised carefully, preventing injury to the nearby plexus. A separate incision was made above the cubital fossa to complete the distal anastomosis.

In a 65-year-old male with triple-vessel disease with normal left ventricular function with near-total occlusion of the left subclavian artery, aorta-axillary artery bypass was done using an 8-mm tubular PTFE graft along with coronary artery bypass graft (CABG). Both procedures were done in the same setting.

Certain tips applied for successful vascular revascularization included aiming for watertight rather than airtight anastomosis and making gentle movements at the level of the wrist of the surgeon rather than at the elbow while taking suture bites, including the adventitia of the vessels while suturing to achieve a better anastomosis. The length and lumen of the graft should be correctly decided to prevent future restenosis.

## Results

A total of 110 patients were operated on for vascular diseases. Among these, 86 (78.81%) were men, and 24 (21.81%) were women. The femoropopliteal segment (n=47) was most commonly involved, followed by the common carotid artery (n=20). Atherosclerosis was the main cause of vascular lesions (81.8%), followed by aneurysm of the aorta (14.5%) and coarctation of the aorta (2.7%). The leading risk factors for the development of the above vascular lesions were smoking (62.2%), followed by hypertension, diabetes, and hyperlipidemia. The majority of patients (92.7%) had a good outcome. Minor complications (7.3%) include seroma formation and wound infection, which were managed conservatively. The repair was performed by autologous vein graft in 30% of patients and by synthetic polytetrafluoroethylene (PTFE) graft in 70% of patients. Carotid artery stenting was the most common endovascular procedure performed (table 2). Femoropopliteal bypass grafting was the most common procedure followed by carotid endarterectomy (n=20) and aortofemoral bypass (n=14). The assessment of outcomes is depicted in Table 4. ABI and abrogation of claudication pain were monitored in peripheral vessel disease, while cardiac/cerebral events were monitored in proximal vessel (carotid and ascending/descending aorta) disease. ABI measured initially before the surgery and ABI measured on follow-up were compared, and improvement in ABI was witnessed in 91.9% of patients in the first month of follow-up, which increased to 98.3% of patients showing improvement in the sixth month. Similarly, 96.9% of patients had abrogation of claudication pain on follow-up. Of the patients who had undergone carotid endarterectomy, three patients suffered from myocardial infarction due to coronary artery disease around five months of the follow-up period. Redo surgery was not required in any of these patients.

**Table 4:** Assessment of outcomes ABI and claudication pain were monitored in occlusive/aneurysmal lesions of peripheral vessels. Cardiac/cerebral events were monitored in occlusive/aneurysmal lesions of proximal vessels (carotid and ascending/descending aorta). ABI: ankle-brachial index; %: percentage of patients

Assessment of outcomes	One month	Three months	Six months
Improvement in ABI	91.9%	96.7%	98.3%
Abrogation of claudication pain	92.3%	95.3%	96.9%
Cardiac/cerebral vascular events	0	0	15%
Redo intervention	0	0	0

## Discussion

The targets of successful management are relief from symptoms, improved quality of life, and diminished risk of cardiovascular and cerebrovascular events. For this, we need to understand the anatomy, collateral network, risk factors, etiology, and pathophysiology.

Anatomy

The wall of the abdominal aorta is thicker and has multiple avascular layers, making it vulnerable to nutrition deficiency and thus degeneration [7,8]. The ratio of iliac artery diameter to the abdominal aorta is 1.15. This ratio reduces to 0.74 by the fifth decade, resulting in increased impedance [9]. This further results in the stretching of the aorta and the formation of atheroma [10]. Two collateral channels develop in aortoiliac occlusive disease (AIOD): systemic and visceral. Systemic-systemic collateral channel involves intercostal, subcostal, lumbar, internal thoracic, superior and inferior epigastric, deep circumflex iliac, iliolumbar median, and lateral sacral and obturator arteries. Visceral-visceral and visceral-systemic channels also exist. The medial pathway is called the arc of Riolan, and the lateral one is Drummond [11]. Occlusions at the distal part of the aorta can be categorized as above, below, or at the level of the inferior mesenteric artery. This signifies the role of the arc of Riolan and the marginal artery of Drummond [12,13]. Therefore, it is essential to identify these collateral pathways before finalizing the management protocol.

Risk factors

Aging is associated with an increased prevalence of PAD [14]. Smoking/tobacco use increases platelet aggregation due to a rise in the production of thromboxane A2, and the lipid profile becomes more atherogenic. These effects are accounted to the nicotine and carbon monoxide components of tobacco. The number and duration of smoking have a strong effect on the extent of the aortoiliac lesion [15]. In population- and epidemiology-based studies, it is estimated that 20%-30% of diabetic patients over 65 years of age have PAD [16]. It is observed that a 26% rise in the risk of atherosclerotic plaque rupture is present with a 1% rise in Hb1Ac [17]. Progression from intermittent claudication to critical limb ischemia occurs at the rate of 1.4% per year. Diabetes results in the thickening of the capillary basement membrane, deranged microanatomy of capillaries, and albumin leak. Infrapopliteal involvement was more common. Asymptomatic cases may develop critical limb ischemia without any significant complaint of claudication. Dyslipidemia results in more proximal aortic lesions [18]. Hypertension, obesity, and syndrome X also contribute. Elevated serum levels of homocysteine have also been documented to be present in atherosclerotic lesions [19].

Management

Diagnostic tests involved X-ray, Doppler studies, digital contrast angiography, multi-detector computed tomography, and magnetic resonance angiography. Treatment should be based on comorbidities, the extent of functional limitations, and the anatomic location of lesions. It involves anti-atherosclerotic therapy, risk factor reduction, and changes in lifestyle before opting for interventional procedures.

Risk factor reduction and lifestyle modification

Total abstinence from smoking is crucial. Medical therapy includes two modalities: exercise and medications. More than 30 minutes per session, >3 sessions per week, and >26-week program is effective in achieving optimum benefit [20]. For the growth of collateral pathways, exercises promoting angiogenesis, vasodilation due to nitric oxide, and enhanced bioenergetics of muscles are responsible for the improvement witnessed.

Anti-atherosclerotic therapy

Antiplatelet therapy (aspirin) helps decrease rates of myocardial infarction and cerebrovascular accidents in such patients [21]. Ramipril has been accounted to reduce the risk of cardiac events by 25% in the above cases [22]. In the clopidogrel versus aspirin in patients at risk of ischaemic events (CAPRIE) trial [23], clopidogrel demonstrated similar benefits. Pentoxifylline, by decreasing blood viscosity and aggregation of platelets, enhances circulation. Cilostazol also prevents platelet aggregation and inhibits smooth muscle cell proliferation, as well as novel therapies such as arterial gene therapy and metabolic agents such as 1-propionyl carnitine and glycoprotein IIb/IIIa receptor antagonist. A team approach with a close working relationship between interventionist and surgeon allows for the setting of clear goals and accepted treatment outcomes.

Interventions

For complex atherosclerotic lesions, two modalities are present to provide revascularization: conventional percutaneous transluminal angioplasty (PTA) and newer percutaneous intentional extraluminal revascularization (PIER). The Peripheral Approach to Recanalization of Occluded Lesions (PATRIOT) trial reported 84% successful crossing of guidewire-resistant chronic total occlusions with Crosser (recanalization catheter) and 0% rate of perforation [24]. Nitinol stents are the most commonly used self-expanding stents. The radial resistive force, good shape memory, and biocompatibility are its advantages [25].

Among grafts for vascular surgery, various options (Dacron versus PTFE), fabrication (knitted versus woven), porosity, and addition of biologic coatings (collagen, gelatin, and albumin) are now available. The size of the graft applied should be according to the type of graft used; for example, Dacron grafts dilate by 10%-20% when exposed to arterial pressures.

Laparoscopic procedures seem to be more cost-effective than conventional open surgery as it is associated with improved quality-adjusted life years (QALYs) and reduced procedure costs. The Covered Endovascular Reconstruction of the Aortic Bifurcation (CERAB) technique shows promising results. Patient selection and familiarity with the technique will influence the outcome. Gene therapy alters the inherent risk of hyperlipidemia, hyperhomocysteinemia, and gene polymorphism. Techniques to enhance therapeutic angiogenesis include injections of DNA encoding vascular growth factors. Catheter-based delivery systems are being developed. Other delivery systems such as infective viruses (e.g., adenovirus), which can incorporate genetic material into the host genome, are being processed.

Limitations of the study

The number of patients undergoing endovascular interventions was less as compared to the surgical group due to the lack of protocol for routine practice of endovascular procedures, causing a mismatch in the number in both groups. The follow-up period included in the study was short.

Similar studies have been conducted comparing the surgical and endovascular procedure outcome for vascular lesions and demonstrate better primary patency with open reconstruction but nearly similar secondary patency, limb salvage, and survival in both groups.

## Conclusions

Our study highlighted the bequest of surgical revascularization. Freedom from re-intervention and minimal complications were witnessed. Novel techniques such as cavo-atrial shunt in Budd-Chiari syndrome provided insight into the utility of surgical revascularization as unconventional therapy. Performing carotid endarterectomy without placing a bypass shunt prompted the role of expertise by which long-term and cost-effective revascularization treatment can be made available. From extensive peripheral arterial disease to proximal vessel lesions, surgical revascularization addresses these successfully.
